# PROGNOSTIC VALUE OF CARCINOEMBRYONIC ANTIGEN LEVELS IN TRANSOPERATIVE PERITONEAL LAVAGE IN PATIENTS WITH GASTRIC CANCER

**DOI:** 10.1590/0102-672020180001e1358

**Published:** 2018-06-21

**Authors:** Letícia Biscaino ALVES, Maria Teresa TSUKAZAN, Ana Elisa SERAFIM, Rolando MENDOZA, Alexandre Vontobel PADOIN, Plínio Carlos BAÚ, Luis Fernando MOREIRA

**Affiliations:** 1 Post-Graduate Program in Surgery, Hospital de Clínicas of Porto Alegre, Federal University of Rio Grande do Sul;; 2 Center for Obesity and Metabolic Syndrome, São Lucas Hospital, Pontifícia Universidade Católica do Rio Grande do Sul (PUCRS);; 3 São Lucas Hospital, PUCRS;; 4 Post-Graduate Program in Medicine and Health Sciences, PUCRS;; 5 School of Medicine, PUCRS, Porto Alegre, RS, Brazil.

**Keywords:** Carcinoembryonic antigen, Peritoneal lavage, Gastric cancer, Peritoneal recurrence, Mortality, Antígeno carcinoembriônico, Lavagem peritoneal, Neiplasia gástrica, Recorrência peritoneal, Mortalidade.

## Abstract

**Background:**

The carcinoembryonic antigen level in peritoneal lavage has been showing to be a reliable prognostic factor in gastric cancer.

**Aim:**

To identify any association between carcinoembryonic antigen level in peritoneal lavage, in gastric cancer patients, with mortality, peritoneal recurrence, tumor relapse or other prognostic factors.

**Methods:**

In total, 30 patients (22 men, 8 women; median age 66 years) with resectable gastric cancer (mainly stage III and IV) were studied. Carcinoembryonic antigen level in peritoneal lavage was detected at operation by immunocytochemical method and a level over 210 ng/g of protein was considered as positive.

**Results:**

There were detected 10 positive cases (33.3%) of plCEA levels. These levels were associated with mortality, RR: 2.1 (p=0.018); peritoneal recurrence, OR: 9.0 (p=0.015); and relapse or gastric cancer progression, OR: 27.0 (p=0.001).

**Conclusion:**

Increased levels of plCEA fairly predicts mortality, peritoneal recurrence tumor relapse or cancer progression.

## RESUMO

**Racional:**

Os níveis do antígeno carcinoembriônico no lavado peritoneal têm sido demonstrados como possível fator prognóstico de recidiva e mortalidade em pacientes com câncer gástrico.

**Objetivos:**

Medir seus níveis em lavado peritoneal durante operação de ressecção de câncer gástrico e ver se eles aumentados estão relacionados com mortalidade, recorrência, recidiva e outros fatores prognósticos.

**Métodos:**

Foi realizado lavado peritoneal durante ressecções de câncer gástrico; os níveis do antígeno carcinoembriônico nesse lavado maiores ou iguais a 210 ng/g foram considerados aumentados ou positivos.

**Resultados:**

Foram estudados 30 pacientes, destes, 33,33 % apresentaram níveis aumentados, os quais foram fator de risco para mortalidade em seis meses OR: 8,5 (1,458-49,539) IC 95%, mortalidade geral RR: 2,111 (1,314-3,391) IC 95%, mortalidade devido à doença OR: 12 (1,885-76,376) IC 95%, recorrência peritoneal OR: 9 (1,325-61,138) IC 95%, e recidiva ou progressão da doença OR: 27 (2,705-269,460) IC 95%.

**Conclusões:**

Os níveis aumentados do antígeno carcinoembriônico no lavado peritoneal foram fatores de risco para mortalidade, recorrência peritoneal, recidiva e progressão da doença em pacientes com câncer gástrico.

## INTRODUCTION

Gastric cancer is the 4^th^ most common cancer in the world^13 )^and occupies the second place in overall cancer mortality^15^. ^)^Although surgical morbidity and mortality decreased in the last 40 years, 5-year survival rate is lower than 30%, in occident^18^.

The peritoneal recurrence is the most common kind of recurrence^17^ ^,^ ^23^, as well as the most common cause of death in patients with gastric cancer^12^ ^,^ ^8^. ^)^When facing serosa-involved cancers, 50% of the patients develop peritoneal recurrence, even after curative surgery^11^ ^,^ ^7^.

Peritoneal lavage cytology is an easy executable method that may indicate a poor survival rate in gastrointestinal carcinomas, due to the fact that it is a well-known cause of peritoneal recurrence^6^.

The sensitivity of cytology in peritoneal lavage is relatively poor, between 22-30% in serosa-involved type of cancers^11^ ^,^ ^5^ ^,^ ^20^ ^,2,^ ^16^. Up to 50% of patients with negative peritoneal cytology results, will develop peritoneal recurrence; whereas up to 20% with macroscopic peritoneal involvement present a negative cytology^23^.

Due to the low sensitivity of conventional cytology, other techniques searching for antigens produced by neoplastic cells have been studied, with the purpose of increasing peritoneal lavage sensitivity.

Carcinoembryonic antigen levels in peritoneal lavage (plCEA) have shown to be good postoperative mortality indicator in serosa-involved gastric cancers, even in those cases without visible peritoneal carcinomatosis during surgery^17^ ^,^ ^4^ ^,^ ^3^ ^,^ ^22^.

Abe et al found positive association between elevated plCEA and serosa invasion. Its elevated levels were independent predictors of gastric cancer mortality and peritoneal recurrence^1^. Wang et al showed the same results in peritoneal recurrence^19^. Only plCEA levels were a significant predictor of mortality in another study. Among patients with peritoneal recurrence, 95% showed positivity^9^.Regardless of the study method used, plCEA levels play an important role as prognostic predictors in gastric cancer patients.

Due to elevated prevalence and the possibility of finding other prognostic factors that could interfere and help on the treatment and survival of patients, peritoneal lavage CEA levels will be measured, analyzing their relation or interference on six month mortality, peritoneal recurrence or general survival, in gastric cancer patients. 

## METHODS

This project was correctly evaluated and approved by the Hospital’s research Ethics Committee, and patients were asked to assign an informed consent. 

Patients with gastric cancer submitted to surgical resection, regardless of the tumor stage, by the general surgery staff at the Hospital São Lucas, Pontifical Catholic University of Rio Grande do Sul, Porto Alegre, RS, Brazil. Were included only the ones that underwent primary tumor resection and peritoneal lavage with adenocarcinoma tumors. The excluding cases were those with urgent indication of surgery that had no proper time for tumor diagnosis or peritoneal lavage; patients with another concomitant digestive cancer, gastric metastasis of another neoplasia; or tumor recurrence after resection surgery.

At the end of the peri-operative period, after laparotomy, 200 ml of physiological saline was introduced into the rectovaginal or the rectovesical spaces; after a manual lavage 20 ml of it was collected for conventional cytology study. Other 20 ml were collected for total protein and CEA levels measuring. The CEA levels were determined with a radiometric immunoassay kit and expressed as ng/g of protein. plCEA levels ≥210 ng/g of protein were defined as positive.

Demographic information, cancer stage pre and post-surgery, comorbidity, signs and symptoms, targeted therapy, disease evolution, and treatment response were evaluated. The patients were follow-up until these study end or death.

### Statistical analysis

All were done with SPSS statistics software. The *χ*2 test was used to analyze the association between the variables. It was used logistic regression to analyze the significate variables in *χ*2 test. The survival rate was calculated by Kaplan-Meier method and statistical difference was evaluated by long-rank test. 

The calculated sample size was 60 gastric cancer patients, considering a mean survival rate of 30% in patients with positive plCEA, which was 10% smaller than overall survival in current literature in a six month period. It was considered as significant a 95% confidence interval and statistic power of 80%.

## RESULTS

Thirty patients with gastric cancer were evaluated, 22 men and eight women, with a median age of 66 years (42-97). They were followed-up in a mean time of 17.43 months (0-46). 

Tumor characteristics are shown on Table 1. Distant metastases were found in 23.3%, from which 57% deceased before being discharged from the hospital. The peritoneal levels of CEA were between 25 ng/g to 21,200 ng/g, with a median of 157.5 ng/g. Peritoneal levels of CEA ≥210 ng/g were found in 33.33% of patients. 

**TABLE 1:** Tumor characteristics of patients with gastric cancer (n=30)

Characteristics	n (%)
Serosa invasion (T3 ouT4)	25 (83.3)
Lymph node’s involvement (N1-3)	20 (66.7)
Distance metastasis (M1)	7 (23.3)
Clinical stage IV	12 (40)
Positive cytology	2 (6.7)
Positive plCEA	10 (33.33)

During the follow-up period 37.6% had cancer relapse, and in 63.6% of them, the peritoneal recurrence was the first kind of relapse. The recurrence in six month was 23.3% (Table 2). 

**TABLE 2:** Recurrence in patients with gastric cancer

Recurrence	n (%)
Recurrence in six months	7 (23.3)
Total recurrence	11 (36.7)
Peritoneal recurrence	7 (23.3)
Recurrence or gastric cancer progression	14 (46.7)

The factors related to elevated plCEA were T4 stage (p=0.015), involvement of lymph nodes (p=0.006), positive cytology (p=0.038) and stage grouping IV (IV SG) (p=0.002, Table 3).

**TABLE 3:** Factors related to elevated plCEA

Factors	Statistical significance
Positive cytology	p=0.038
Involvement of lymph nodes	p=0.006
T4 stage	p=0.015
IV SG	p=0.002

The plCEA levels were the only risk factor to peritoneal recurrence, with OR: 9 (1.325 - 61.138) 95CI, p= 0.015. 

Positive plCEA levels, involvement of lymph nodes, IV SG, T4 stage, distant metastases, residual disease, extended lymphadenectomy not performed, and lack of adjuvant treatment were risk factors for tumor relapse or disease progression. The plCEA levels ≥210 ng/g were the only independent risk factor for tumor relapse or disease progression (Table 4).

**TABLE 4:** Factors related to recurrence or disease progression

Factors	Univariate analysis	Logistic regression
plCEA	OR: 27 (2.705 - 269.460)95 IC	OR: 38.206 (1.075 - 1358.419)95 IC NS* NS*
Lymph nodes involvement	OR: 16.714 (1.742 - 160.350)95 IC	
T4 stage	OR: 11.25 (1.146 - 110.461)95 IC	
Distant metastases	OR: 11,25 (1,146 - 110,461)95 IC	NS*
IV stage grouping	OR: 17.5 (2.667 - 114.846)95 IC	NS*
Residual disease	RR: 1.4 (1.005 - 1.950)95 IC	NS*
Extended lymphadenectomy not performed	OR: 5.4 (1.120 - 26.044)95 IC	NS*
Adjuvant therapy not performed	OR: 6 (1.003 - 35.908)95 IC	NS*

*Not significant

**FIGURE 1:**
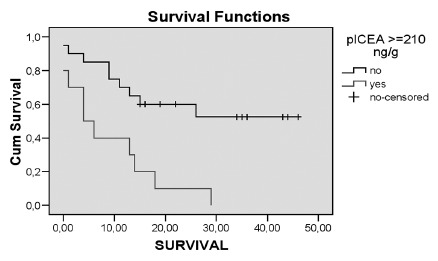
Overall survival

Was found 30% mortality in a six month period, which correlated with positive plCEA levels, residual disease and lack of adjuvant therapy (Table 5). The overall survival was 36.7%, significantly lower in patients with positive plCEA levels (Figure 1). Regarding to overall mortality, positive plCEA levels, resection without extended lymphadenectomy, distant metastases, lymph node involvement, T4 stage, IV SG and lack of adjuvant therapy were correlated factors (Table 5). In concern to disease mortality during follow-up, the related factors were positive plCEA levels OR: 12 (1.885 - 76.376)95 IC, lymph node involvement OR: 13.5 (1.421 - 128.258)95 IC, T4 stage OR: 13.714 (1,381 - 136,212)95 IC and IV SG OR: 10.5 (1.885 - 58.359)95 IC.

**TABLE 5:** Factors related to mortality

	Mortality in six months		General mortality	
Factors	Univariate analysis	Logistic regression	Univariate analysis	Logistic regression
plCEA	OR: 8.5 (1.458 - 49.539)IC 95%	NS*	RR: 2.111 (1.314 - 3.391)IC 95%	NS*
Residual disease	RR: 1.8 (1.003 - 3.229)IC 95%	NS*	NS*	NA**
Lymph node involvement	NS*	NA**	OR: 9.333 (1.637 - 53.208)IC 95%	NS*
Stage T4	NS*	NA**	RR: 1.583 (1.123 - 2.232)IC 95%	NS*
IV SG	NS*	NA**	RR: 2.714 (1.507 - 4.890)IC 95%	NS*
Distant metastases	NS*	NA**	RR: 1.583 (1.123 - 2.232)IC 95%	NS*
Extended lymphadenectomy	NS*	NA**	OR: 6.188 (1.041 - 36.779)IC 95%	NS*
Lack of adjuvant therapy	RR: 1.818 (1.223 - 2.703)IC 95%	NS*	OR: 22.667 (3.140 - 163.629)IC 95%	NS*

*No significance **Not available

## DISCUSSION

The importance of this study is related to the current need to find, among gastric cancer patients, the ones with worse prognostic factors, so they can be provided with an appropriate adjuvant therapy.

Were found results similar to previous studies, showing a positive association between elevated plCEA levels and tumor recurrence^21,1,19 )^and mortality^4,3,1,9 )^in patients who underwent tumor resection. What´s more, this is the first western study to confirm these results, probably because we used the same methods and cut points of previous studies**.**

Some researchers have not found any relation between plCEA levels and peritoneal recurrence or survival, but their peritoneal washing technique included at least 600 ml saline solution with no correction by protein levels. So, it is difficult to compare their findings to ours or to others that used plCEA levels in ng/g of protein^17^ ^,^ ^4^ ^,^ ^3^ ^,^ ^1^ ^,^ ^19^ ^,9,^ ^10^.

Was also found plCEA positive levels in a higher percentage compared to studies which used a similar cut point^1^ ^,^ ^19^, probably because our patients had more advanced tumor stages. This enforces the fact that there are other important prognostic factors beyond the already well-established ones, which are vital to define the kind of treatment offered, especially to advanced tumor patients. 

In the multivariate analysis these findings didn’t show statistical significance, probably because the number of patients was not enough to differentiate groups.

Although was not reached initial number, was found statistical significance in relation to mortality in a six month period, probably because the great difference in death rate between groups, being 60% in plCEA >=210 and 15% in plCEA <210 ng/g.

Even with such a small number, the mortality difference between groups cannot be ignored, being 80% in plCEA positive and 25% in plCEA negative patients during disease follow-up, and 90% vs. 45% overall mortality, respectively. Neither can we randomly attribute these differences to the fact that patients with T4 stage with lymph node involvement or IV SG have positive plCEA levels, since our number of patients was not enough to distinguish groups. 

We should, however, analyze and compare our results with others in current literature, considering plCEA levels as an important risk factor to peritoneal recurrence and mortality.

Possible reasons for peritoneal recurrence could be either the existence of cancer cells in the abdominal cavity due to gastric wall invasion prior to surgery or the release of neoplastic cells by lymph vessels sectioned during surgery, or even by iatrogenic dissemination caused by the surgical act itself^14^. The presence of cancer cells in peritoneal cavity, that produce CEA, but are not detected by conventional cytology, could explain the relation between plCEA levels and peritoneal recurrence.

The most reliable hypothesis for elevated CEA levels in peritoneal wash is that cancer cells produce a sufficient amount of CEA to be detected^1^. This could explain the correlation between CEA peritoneal levels and T4 stage, but not the association with lymph node involvement.

Another mechanism would be the liberation of elevated CEA levels in the peritoneal cavity by elevated blood levels of CEA^1^.

To define whether CEA levels are produced by neoplastic cells inside the peritoneal cavity or by the primary tumor, more accurate studies involving this issue are needed. However, we should at least consider the possibility that plCEA levels are more sensitive than conventional cytology to detect neoplastic cells or to determine the risk of peritoneal recurrence. 

In this study tumor invasion of other organs and positive cytology were linked to elevated CEA levels, a finding that could correlate wall invasion with plCEA levels. However, other factors associated to plCEA, like lymph node involvement and IV SG, are not necessarily related with invasion depth. Besides, we haven’t found any relation between T3 stage and elevated CEA levels. Consequently, we don’t have enough evidence-based data to speculate the mechanisms involved with elevated CEA levels in the peritoneal cavity. 

Most of the patients diagnosed with gastric cancer that undergo surgical resection in western countries show more advanced stages of the disease and hence worse survival rates, which reinforces the need to use other prognostic factors as well as new therapies to offer them. 

Peritoneal lavage levels of CEA were discovered a couple of years ago and are being used as indicators of peritoneal recurrence, one of the most common kinds of tumor relapse. In this study elevated plCEA levels were the only significant prognostic factor for developing peritoneal recurrence, as well as the only risk factor for relapse or disease progression in multivariate analysis.

The peritoneal wash is undertaken in the beginning of the surgery, before tumor manipulation, being the results available within 1-3 h depending on the method, resulting in information that can influence the decision of therapeutic options. 

Forthcoming studies will probably search the utilization of new therapies specifically developed to avoid peritoneal recurrence (such as transoperative chemotherapy) in patients more susceptible to this kind of recurrence (like the ones with elevated plCEA levels).

While new therapies are not yet well-established, plCEA levels can be helpful to decide whether performing extended lymphadenectomy during surgery or postoperative chemo and radiotherapy. 

Bringing to a close, the results shown suggest that the use of carcinoembryonic antigen level in peritoneal lavage does determine and may be used as a reliable predictive factor of worst prognosis, resulting on a useful tool when deciding for the best and most accurate treatment. 

## CONCLUSIONS

Elevated CEA levels are significantly associated with a higher mortality rate inside the first six months after resectional surgery. They are also directly related to lymph involvement, transmural invasion and an advance clinical stage. Higher levels of CEA are greatly associated with general mortality and disease-related mortality, as well as tumor progression and tumor recurrence. It is important to take into consideration that the increase of CEA levels in peritoneal lavage is the only significant factor associated with peritoneal recurrence.
